# Marine-Derived Bioactive Ingredients in Functional Foods for Aging: Nutritional and Therapeutic Perspectives

**DOI:** 10.3390/md22110496

**Published:** 2024-11-04

**Authors:** Youngji Han, Dong Hyun Kim, Seung Pil Pack

**Affiliations:** 1 Biological Clock-Based Anti-Aging Convergence RLRC, Korea University, Sejong-ro 2511, Sejong 30019, Republic of Korea; youngjihan@korea.ac.kr; 2 Department of Biotechnology and Bioinformatics, Korea University, Sejong-ro 2511, Sejong 30019, Republic of Korea; jklehdgus@korea.ac.kr

**Keywords:** aging, anti-aging, functional food, marine-derived bioactive compounds

## Abstract

Aging is closely linked to various health challenges, including cardiovascular disease, metabolic disorders, and neurodegenerative conditions. This study emphasizes the critical role of bioactive compounds derived from marine sources, such as antioxidants, omega-3 fatty acids, vitamins, minerals, and polysaccharides, in addressing oxidative stress, inflammation, and metabolic disorders closely related to aging. Incorporating these materials into functional foods not only provides essential nutrients but also delivers therapeutic effects, thereby promoting healthy aging and mitigating age-related diseases. The growth of the global anti-aging market, particularly in North America, Europe, and Asia, underscores the significance of this study. This review systematically analyzes the current research, identifying key bioactive compounds, their mechanisms of action, and their potential health benefits, thus highlighting the broad applicability of marine-derived bioactive compounds to enhancing healthy aging and improving the quality of life of aging populations.

## 1. Introduction

The aging process is characterized by a complex interplay of genetic, environmental, and lifestyle factors, leading to physiological, functional, and aesthetic changes over time [1]. As the global population continues to age, the extension of a healthy lifespan and the mitigation of age-associated diseases and functional decline are increasingly being emphasized [2]. Anti-aging efforts encompass multiple strategies, including lifestyle modifications, medical treatments, and innovative technologies [3,4,5]. Among these strategies, the use of marine-derived bioactive compounds in functional foods has emerged as a promising area of research with significant potential for addressing age-associated diseases.

Marine environments constitute a rich source of bioactive compounds that possess unique structures and potent biological activities [6,7]. These compounds have demonstrated potential in combating various metabolic disorders, such as oxidative stress, inflammation, metabolic disorders, and immune system decline [8,9]. The incorporation of marine-derived materials into functional foods offers the dual benefit of providing essential nutrients while delivering therapeutic effects, rendering them an attractive component of anti-aging diets [10,11].

**Figure 1:**
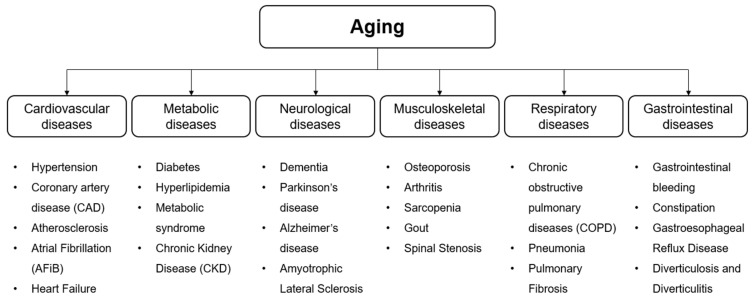
Aging-related disease.

Age-associated diseases often share common characteristics, such as a chronic nature, the co-existence of multiple conditions, functional decline, and a reduced capacity for recovery [12,13]. As shown in Figure 1, the major categories of age-associated diseases encompass cardiovascular (CVD; e.g., hypertension, coronary artery disease, etc.), metabolic (e.g., diabetes, hyperlipidemia, etc.), neurological (e.g., dementia, Parkinson’s disease, etc.), musculoskeletal (e.g., osteoporosis, arthritis, etc.), respiratory (e.g., chronic obstructive pulmonary disease, pneumonia, etc.), and gastrointestinal (e.g., gastrointestinal bleeding, constipation, etc.) diseases. These conditions significantly impact quality of life in older adults and present considerable challenges to healthcare systems.

The global anti-aging market is experiencing rapid growth, driven by technological advancements and a rising interest in personalized health solutions [14]. North America and Europe currently lead in anti-aging research and product development, while Asia, particularly South Korea and Japan, is witnessing a precipitous expansion of the anti-aging sector owing to its aging populations and increased health awareness. In this context, marine-derived bioactive compounds present a novel and promising avenue for the development of functional foods aimed at promoting healthy aging and managing age-associated diseases.

**Figure 2:**
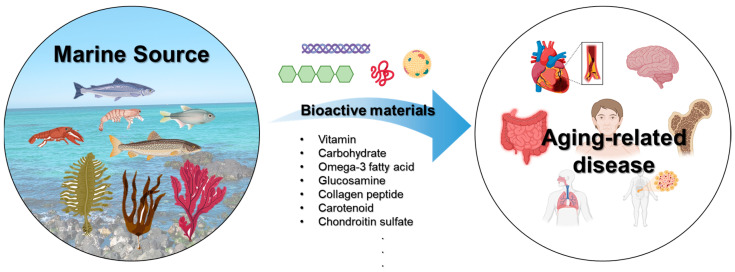
Schematic representing functional foods and therapeutic bioactive compounds derived from marine sources targeting aging-related diseases.

This review article explores the nutritional and therapeutic potential of safe and consumable health functional food materials derived from various marine sources (Figure 2). It examines the current state of the research, highlights the key bioactive compounds involved, and discusses their mechanisms of action and potential health benefits. This review aims to provide a comprehensive overview of the role of integrating these materials into functional foods in supporting healthy aging and addressing age-associated diseases, thereby contributing to the broader field of anti-aging research and health promotion.

## 2. Antioxidant Activity

Although the marine environment offers a wealth of commercially valuable natural products with antioxidant properties, it remains largely underutilized [15]. A wide range of marine organisms, from seaweeds and sponges to bacteria and fungi, significantly contribute to natural antioxidant production [16]. The antioxidant potential is influenced by factors such as the species, the extraction and purification processes, and the environmental conditions in which the marine organisms grow, leading to compositional variations within the same species globally [17].

### 2.1. Antioxidant Vitamins

Marine-derived vitamins with antioxidant properties play a crucial role in protecting cells from oxidative stress [18]. Antioxidant vitamins, such as vitamins C, E, and A, safeguard cellular integrity by neutralizing free radicals, inhibiting lipid peroxidation, regenerating oxidized antioxidants, mitigating oxidative stress, and modulating the expression of genes associated with antioxidant defense mechanisms [19,20,21]. Seaweed is rich in vitamin C. The minimal differences observed among various seaweed types suggest that the vitamin C levels are generally similar across different species of seaweed, regardless of their taxonomic classification [22].

Vitamin E, a fat-soluble antioxidant, protects cells from oxidative damage by neutralizing free radicals, especially within lipid membranes [23]. It acts synergistically with other antioxidants, such as vitamin C, and is essential for maintaining cellular health [24,25]. Its antioxidant properties may help reduce the risk of chronic disease. The marine microalgae *Dunaliella tertiolecta* and *Tetraselmis suecica* actively produce alpha-tocopherol [26].

### 2.2. Selenium

Selenium is a trace mineral that plays an indispensable role in the body’s antioxidant defense system. Its antioxidant capacity primarily arises from its function as a cofactor for various enzymes, such as glutathione peroxidase, thioredoxin reductase, and deiodinase, particularly those involved in protecting cells from oxidative stress. In nature, organic selenium primarily occurs in the following forms: SeCys and SeMet [27]. Crabs (*Callinectes sapidus*), oysters (*Crassostrea virginica*), shrimp (*Penaeus duorarum*), and Baltic herring (*Clupea harengus*) are rich marine sources of selenium [28]. Zhen Xia et al. reported that selenium-enriched antioxidant peptides, purified from selenium-enriched oyster hydrolysate, possess strong antioxidant properties, such as inhibiting reactive oxygen species (ROS) production and enhancing antioxidant enzyme activity [29].

## 3. Marine Compounds for CVD

CVD remains a major cause of morbidity and mortality globally, with its prevalence rising significantly in recent decades. In 2021, CVD was responsible for approximately 20.5 million deaths, up from 12.1 million in 1990, reflecting the impacts of global population growth and aging (American College of Cardiology; World Heart Federation). Furthermore, an estimated 523 million people worldwide were living with CVD in 2020, highlighting the extensive scope of this health issue (CDC). CVD accounts for 32% of all global deaths, with a disproportionate number occurring in low- and middle-income countries (World Heart Federation). These alarming statistics underscore the urgent requirement for effective prevention and management strategies to combat the growing CVD burden and enhance global health outcomes. In response to this concern, marine sources have garnered significant attention in the pharmaceutical and health food industries. This review provides an overview of marine resources, bioactive compounds, CVD-related diseases, and biological effects (Table 1).

### 3.1. Omega-3 Fatty Acids

The increased integration of omega-3 fatty acids into functional foods, driven by their considerable health benefits, has closely intertwined the omega-3 fatty acid and functional food markets. The global omega-3 fatty acid market, which includes both supplements and functional foods, was valued at approximately USD 2.49 billion in 2019 and is projected to expand at a compound annual growth rate of 7% from 2020 to 2027 [41].

Omega-3 fatty acids, especially eicosapentaenoic acid (EPA) and docosahexaenoic acid (DHA), are predominantly found in various types of fatty fish and seafood (e.g., salmon, mackerel, herring, trout, and algae) and are readily absorbed and utilized by the human body. They exert numerous beneficial effects on CVD [42]. Elevated triglyceride levels constitute a significant risk factor for CVD, and their reduction is crucial for cardiovascular health. The regular consumption of marine omega-3 fatty acids has been shown to reduce blood triglycerides by inhibiting diacylglycerol O-acyltransferase.

Chronic inflammation plays a critical role in the development of atherosclerosis. EPA and DHA mitigate inflammation by reducing the production of pro-inflammatory eicosanoids derived from arachidonic acid and enhancing the generation of anti-inflammatory eicosanoids and resolvins [43,44]. This anti-inflammatory effect helps prevent plaque formation and progression within arteries [45]. Endothelial dysfunction, a precursor to atherosclerosis and hypertension, is positively influenced by marine-derived omega-3 fatty acids through enhanced nitric oxide (NO) production, improved vasodilation, and increased blood flow [46].

The stabilization of atherosclerotic plaques is requisite to averting acute cardiovascular incidents. Omega-3 fatty acids enhance plaque stability by reducing the inflammation within plaques, thereby alleviating the risk of plaque rupture, which can result in myocardial infarction or stroke [45]. Furthermore, omega-3 fatty acids improve endothelial function and promote vasodilation, contributing to modest blood pressure reductions [47]. Since hypertension is a major CVD risk factor, managing it is essential for overall cardiovascular health. The Reduction of Cardiovascular Events with Icosapent Ethyl-Intervention Trial (REDUCE-IT) is a landmark clinical trial aimed at evaluating the efficacy of icosapent ethyl, a purified form of the omega-3 fatty acid EPA, in mitigating cardiovascular events in high-risk patients [48]. This trial included participants with established CVD or diabetes and additional risk factors who had fasting triglyceride levels between 135 and 499 mg/dL. These participants were administered icosapent ethyl at a dosage of 4 g/day. REDUCE-IT yielded compelling evidence that a daily intake of 4 g of icosapent ethyl significantly lowers the risk of major cardiovascular events in high-risk patients with elevated triglycerides, even when they are on statin therapy. Additionally, several clinical studies have demonstrated that omega-3 fatty acids improve CVD outcomes. As such, omega-3 fatty acids have gained prominence not only in CVD clinical research but also in other diseases of aging, making them one of the most powerful bioactive substances available today.

### 3.2. Zeaxanthin

Zeaxanthin is a xanthophyll carotenoid that is found in algae or microalgae. Furthermore, research is being conducted to identify novel extraction methods, including ultrasound-based, microwave-based, and UV/MS-based techniques, along with the development of new microalgae and marine species (e.g., *Tisochrysis lutea*, *Porphyridium cruentum*, and *Phaeodactylum tricornutum*), for the purpose of obtaining zeaxanthin [49,50,51]. Zeaxanthin displays significant promise in the prevention and management of CVD through its antioxidant, anti-inflammatory, and endothelial-function-improving properties [52]. El-Baz, F.K. et al. demonstrated the effect of zeaxanthin, which was extracted from *Dunaliella salina*, on cardiac dysfunction. Zeaxanthin reduced the cardiac level of interleukin-6 (IL-6), inducible nitric oxide synthase (iNOS), superoxide dismutase (SOD), and nuclear factor kappa B (NF-κB) in cardiac dysfunction induced in rats. Oral administration of zeaxanthin (250 µg/kg) to the rats resulted in the restoration of their cardiac SOD content to values that were nearly normal and an improvement in their cardiac NF-κB levels by approximately 18%. The gene expression of retinoic acid receptor alpha (RAR-α) is associated with cardiac dysfunction. The rats with cardiac dysfunction showed a significant downregulation of RAR-α. However, their gene expression of RAR-α was recoveredby up to 77% after the oral adminstration of zeaxanthin [30].

Low-density lipoprotein cholesterol (LDL-C) is recognized as a critical factor in the development of CVD. Specifically, small dense LDL particles and oxidized LDL are strongly associated with an increased risk of atherosclerosis, which can lead to severe cardiovascular events, such as heart attacks and strokes [53]. However, zeaxanthin has been shown to reduce the oxidation of these LDL particles, potentially reducing the onset and progression of CVD [54]. In the study by Dwyer, J.H et al., they confirmed a relationship between zeaxanthin and carotid intima–media thickness (IMT) in a random cohort of 269 women and 304 men [55,56]. In various regulatory studies, zeaxanthin has demonstrated high safety. Zeaxanthin did not cause toxicity or teratogenicity in rats or rabbits at doses up to 1000 mg/kg and 400 mg/kg body weight/day, respectively. In humans, the EU Commission has approved a use level of 2 mg per day [57].

### 3.3. Alginate Oligosaccharides

Alginate oligosaccharides are bioactive substances that have been approved by the United States Food and Drug Administration (FDA). Alginate oligosaccharides are extracted primarily from marine algae, which are commonly referred to as brown seaweed [58]. Brown seaweed is a readily cultivated marine organism, and the alginate oligosaccharides derived from it exhibit high chemical stability and biocompatibility, rendering them suitable for a multitude of medical and industrial applications [59]. The extraction process typically entails alkaline treatment of the algae to isolate the alginate, followed by purification and concentration to obtain the desired form of the alginate oligosaccharides [60,61]. The alginate oligosaccharides obtained through this process have many uses in biomedical applications, as thickening agents, as dental impression materials, and for gel formation [62,63].

Alginate oligosaccharides have also shown potential to prevent the development of CVD. Alginate oligosaccharides can significantly reduce both systolic blood pressure (SBP) and mean arterial pressure (MAP) [62]. The anti-inflammatory properties of alginate can reduce vascular inflammation, leading to improved vascular elasticity. The short-chain fatty acids produced during the fermentation of alginate oligosaccharides exerted a protective effect against CVD by enhancing endothelial cell function and reducing inflammatory processes [64]. In addition, alginate has the potential to improve overall cardiovascular health by inhibiting sodium-glucose co-transporter 1 (SGLT-1) in the kidneys, which may result in increased sodium excretion [65]. Yi Hu et al. demonstrated that alginate oligosaccharide restrained the activation of the P-selection/p38MAPK/NF-κB pathway in monocrotaline (MCT)-induced pulmonary hypertension in rats. In addition, the administration of increasing concentrations of alginate oligosaccharide to the rats (5 mg/kg/day, 10 mg/kg/day, and 20 mg/kg/day) yielded results indicative of a reduction in pulmonary arterial wall area and wall thickness [37]. In a study by Wang et al., alginate oligosaccharides activated the glutathione peroxidase 7 (GPX7) pathway through the intranuclear translocation of Nrf2, which could delay vascular aging by reducing oxidative stress levels and increasing antioxidant properties in older rats [66].

## 4. Marine Compounds for Skin Aging

**Figure 3:**
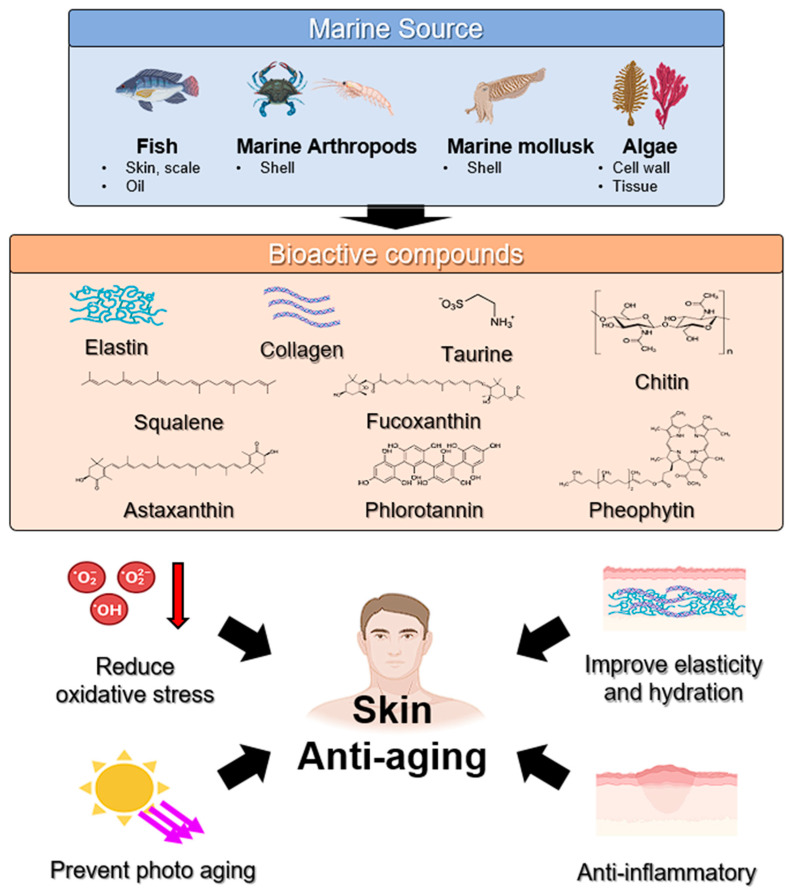
Skin anti-aging using marine-derived bioactive compounds.

Prolonged exposure to diverse external factors, such as physical force, chronic light exposure, pollution, and chemicals, alongside internal factors, including genetics, hormonal regulation, and metabolic processes, cumulatively leads to the complex biological phenomenon known as skin aging [67]. Internal aging occurs naturally and is marked by decreased skin elasticity, a rougher skin texture, and pronounced wrinkles. In contrast, external aging is driven by environmental elements, notably ultraviolet light, ROS, and stress [68,69]. Marine resources constitute an optimistic and eco-friendly source of unique bioactive substances for the cosmetic sector, presenting promising solutions for mitigating the effects of skin aging. Figure 3 is an illustration of marine-derived compounds with anti-aging properties.

### Marine-Algae-Derived Carbohydrates

Marine algae comprise various substances, including carbohydrates, lipids, proteins, amino acids, minerals, and flavonoids [67,70]. Carbohydrates are the most abundant constituents of marine algae. Marine-algae-derived carbohydrates have been utilized in cosmeceutical industries owing to their chemical and physical properties.

The primary issue in skin aging is the increased expression of matrix metalloproteinases (MMPs), which are involved in collagen synthesis and degradation owing to light exposure [71]. Therefore, the key strategy to address this problem is to reduce MMP levels and control collagen secretion. Fucoidan, a major sulfated polysaccharide found in the cell walls of brown algae [72], has been observed to suppress UVB-stimulated mRNA and protein expression related to MMP-1 regulation and type 1 pro-collagen downregulation by inactivating extracellular signal-regulated kinase (ERK) and c-Jun N-terminal kinase in HaCaT immortalized human keratinocyte cells [73]. Moreover, fucoidan from *Mekabu* inhibited the interleukin-1β (IL-1β)-induced secretion of MMP-9 and MMP-3 and the degradation of tissue inhibitor of metalloproteinases 1 in HDFs [74]. Laminaran is a storage polysaccharide extracted from brown algae [75]. Laminaran from *Saccharina longicruris* reportedly ameliorated UVA/UVB-induced skin dermal thickness via MMP-1 suppression [76]. Carrageenan, a polysaccharide derived from red seaweeds (i.e., *Eucheuma* spp., *Chondrus crispus* [Irish moss], and *Gigartina stellata*), is widely used in food and medicine and as an excipient in cosmetics and skincare products. The three main types of commercially available carrageenan are kappa (κ; forms strong, rigid gels in the presence of potassium ions), iota (ι; forms soft, clear, and elastic gels in the presence of calcium ions), and lambda (λ; does not form a gel and is normally used to thicken dairy products) [77]. Haema et al. reported that ι- and κ-carrageenan prevent collagen breakdown and reduce MMP-1 levels. A κ-carrageenan–collagen peptide complex significantly mitigated UV-induced cell death and apoptosis in HaCaT and MEF cells by reducing intracellular ROS levels. The complex inhibited UV-induced type 1 pro-collagen reduction and MMP-1 elevation by suppressing the mitogen-activated protein kinase signaling pathway [78]. Discoloration, which serves as a biomarker of skin aging, is related to the presence of melanin [79]. Dark spots emerge due to an excessive and irregular distribution of melanin in the skin. Melanin is produced under the influence of tyrosinase during melanogenesis, and its levels vary with age, possibly because of menopause [79]. Fucoidan suppressed melanogenesis by activating the ERK pathway in Mel-Ab cells, while fucoidan treatment did not directly decrease tyrosinase activity [80]. Carrageenan from red algae effectively degraded and eliminated dermal melanosomes and melanin from the dermis of guinea pigs, indicating the skin-whitening potential of carrageenan [81].

## 5. Marine Compounds for Bone and Joint Health

**Figure 4:**
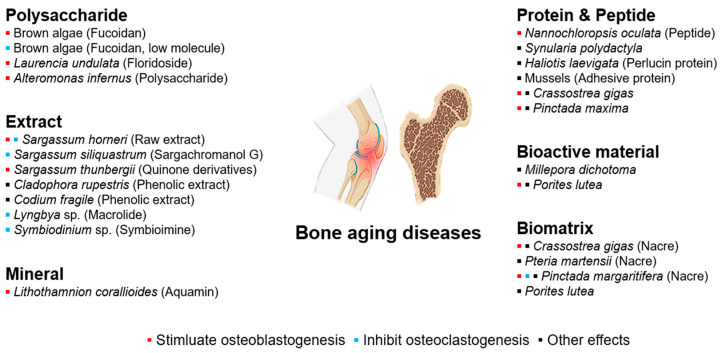
Bioactive compounds from marine sources protective from bone and joint aging diseases.

As humans age, bone tissue is the first to exhibit signs of aging. The aging of bone is specifically characterized by a gradual reduction in bone mass and bone density over time [82]. Age-related bone diseases include osteoporosis [83], osteoarthritis [84], rheumatoid arthritis [85], and periodontitis [86]. Treating bone defects and diseases related to aging presents a multifaceted challenge that requires various approaches and techniques. Bone therapies typically focus on three fundamental properties: cellular components, scaffolds for tissue architecture, and bioactive or growth factors [87,88]. While cellular components are essential, scaffolds and bioactive compounds can be sourced from various fields. Notably, there is growing interest in the use of scaffolds and bioactive compounds, particularly those derived from marine organisms, to address the challenges of bone aging [89,90,91]. Figure 4 shows marine compounds that affect age-related diseases of the bones and joints.

### 5.1. Osteoporosis

Osteoporosis, the most common metabolic bone disease and characterized by a low bone mineral density and reduced bone strength, significantly increases fracture risk and is a major health concern, especially for the aging population. In 2010, more than 99 million adults aged ≥ 50 years old were estimated to have a severely decreased bone density mass in the United States [92]. Based on an overall osteoporosis prevalence of 10.3%, 10.2 million older adults (age: ≥65 years old) were estimated to have osteoporosis in the United States in 2010. The overall prevalence of a low bone mass was 43.9%, and 43.4 million older adults were estimated to have mild to severe levels of osteopenia. In the European Union, the prevalence of densitometric osteoporosis, defined by a T-score ≤ −2.5 at the femoral neck, was estimated at 6% and 47% in European women aged 50–55 and ≥80 years old, respectively, and 2.5% and 16% in European men of the same age, respectively [93].

#### 5.1.1. Marine-Algae-Derived Minerals

Hydroxyapatite, a naturally occurring mineral form of calcium apatite, is vital for bone regeneration owing to its biocompatibility [94]. It supports osteoblast attachment, proliferation, and differentiation, all of which are imperative for new bone formation [95,96]. Frequently used in bone regeneration, hydroxyapatite, derived from non-marine sources, is valued for its excellent osteoconductivity and resemblance to bone minerals [97]. However, acknowledging that the predominant inorganic component of human bone is significantly different from pure hydroxyapatite is important [98]. The adaptability of hydroxyapatite increases when it is sourced from biological materials such as fish bones or marine biogenic calcium carbonates commonly present as calcite or aragonite [99]. Hydroxyapatite obtained from *Katsuwonus pelamis* enhanced osteogenic proliferation and differentiation in dexamethasone-treated MC3T3-E1 osteoblasts [100].

#### 5.1.2. Aquamin^®^

Aquamin is a natural, multi-mineral supplement derived from the red marine algal species *Lithothamnion* [101]. It is rich in highly bioavailable calcium, magnesium, and over 70 other trace minerals and is easily absorbed and utilized by the body. Aquamin treatment promotes increased mineralization in osteoblast cell culture [101]. Additionally, 20-week Aquamin treatment resulted in less deterioration of the trabecular bone structure and improved mineral composition and tissue-level biomechanical properties in rat tibia following ovariectomy compared to calcium carbonate treatment in female retired breeder Wistar rats [102].

### 5.2. Osteoarthritis

Osteoarthritis is the most prevalent form of arthritis and a major cause of chronic disability in older adults [103]. Nonsteroidal anti-inflammatory drugs are frequently used to treat osteoarthritis; however, they can cause serious gastrointestinal and cardiovascular side effects, and they do not exert any protective effects on the cartilage beyond symptom relief [104,105]. Therefore, to treat osteoarthritis, medications that not only alleviate its symptoms but also prevent or mitigate the progression of cartilage damage are required.

#### 5.2.1. Glucosamine

Glucosamine, an amino-saccharide prevalent in the cartilage and synovial fluid, serves as a crucial substrate for the biosynthesis of glycosaminoglycan chains, aggrecan, and various proteoglycans within the cartilage [106]. Notably, aggrecan’s hydrophilic properties positively contribute to the management of osteoarthritis, enhancing joint function and integrity [107]. Traditionally, glucosamine has been sourced from the shells of marine shellfish, including shrimp, crab, crawfish, prawns, squid pens, and krill [108]. In recent developments, algae-derived glucosamine has been actively pursued as a vegan alternative suitable for individuals with shellfish allergies, thereby mitigating the risks associated with shellfish-derived glucosamine [109].

Glucosamine sulfate (GS) demonstrates significant anti-inflammatory properties by reducing prostaglandin E2 synthesis in chondrocytes and synovial membrane cells, thus inhibiting superoxide radical production and suppressing lysosomal enzyme activity, as well as inducible NO synthesis [110]. Furthermore, GS facilitates proteoglycan synthesis while curtailing the activity of catabolic enzymes, such as collagenase [111]. This anti-catabolic action underpins the therapeutic efficacy of glucosamine, with GS exhibiting a more pronounced effect than glucosamine hydrochloride [112].

#### 5.2.2. Chondroitin Sulfate

Chondroitin sulfate (CS), a major component of the extracellular matrix in cartilage, bones, skin, ligaments, and tendons, is notably present in the cartilage and fins of diverse shark species [113]. In joint cartilage, CS contributes to the creation of osmotic swelling pressure within aggrecan [114], thus expanding the matrix and supporting the formation of a collagen network [115]. Additionally, it exhibits anti-inflammatory properties, promotes proteoglycan synthesis, and inhibits proteolytic enzymes that degrade the cartilage matrix and induce chondrocyte apoptosis, thereby mitigating catabolic processes [116,117,118].

## 6. Marine Compounds for Sarcopenia

**Figure 5:**
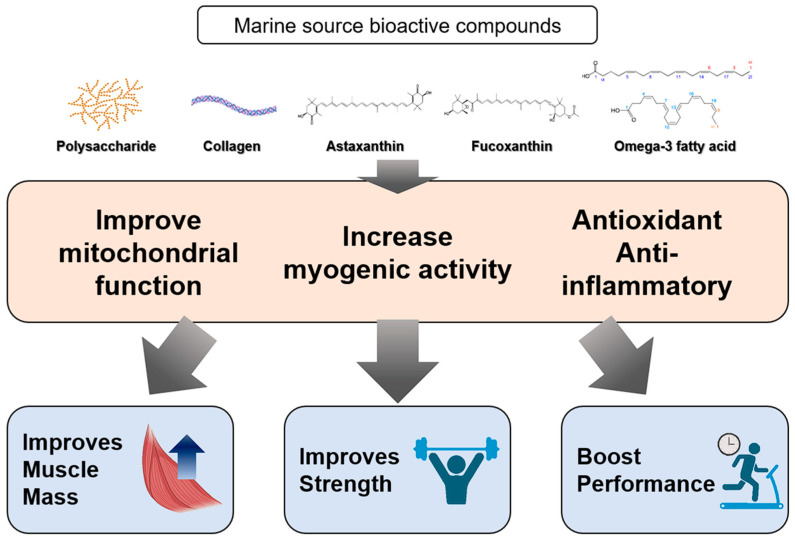
Main biological effects of marine compounds and their expected effects.

As individuals age, they may experience sarcopenia, a condition marked by a gradual decrease in muscle mass, strength, and function. Annually, older adults lose approximately 1% of their muscle mass and strength [119]. This process, which generally commences in middle age and intensifies after 60, is influenced by hormonal changes, reduced physical activity, poor dietary habits, and chronic inflammation. Considering the imperative of uncovering new substances for the treatment and prevention of age-related sarcopenia, marine sources have increasingly been examined for their potential role in addressing this condition, a phenomenon attributed to their high nutrient and bioactive compound content (Figure 5).

### 6.1. Collagen Peptide

Collagen is a major component of the connective tissue, including muscle, tendons, and ligaments, and it plays a significant role in muscle generation and overall muscle health. Dietary collagen supplementation contributes to increased muscle weight and improved muscle function through the support of muscle structure and the provision of amino acids with anti-inflammatory effects. Fish skin and scales are excellent marine sources of collagen [120] known for their high bioavailability and effectiveness because of their smaller peptide molecules compared with those of other collagen sources. The insulin-like growth factor 1 (IGF-1)/phosphoinositide 3-kinase (PI3K)/protein kinase B (Akt) signaling pathway enhances protein synthesis via mammalian target of rapamycin activation and reduces protein degradation by suppressing forkhead box transcription factor class O transcription factors, thereby preventing the upregulation of muscle-atrophy-related genes. Collagen derived from catfish skin gelatin mitigated aging-related sarcopenia by activating the IGF-1/PI3K/Akt signaling pathway in middle-aged mice [121]. Additionally, in a fracture mouse model, type 2 collagen from squid cartilage induced myogenic IGF-1 and irisin, which are myokines related to muscle growth and development and muscle cell production, respectively [122]. Consistent with these results, a clinical study found post-exercise protein supplementation with collagen peptides to significantly affect muscle mass and function compared with the placebo following resistance training in older patients with sarcopenia [123]. Consequently, collagen peptides have exhibited favorable outcomes in clinical trials and are currently the focus of extensive research.

### 6.2. Marine Carotenoids

Fucoxanthin is a marine carotenoid specifically produced by brown algae and diatoms. It exerts several beneficial effects on obesity, cancer, and oxidation. In studies of dexamethasone-induced atrophy in C2C12 myotubes, fucoxanthin demonstrated preventive effects by improving protein proteolysis, mitochondrial function, autophagy, and apoptosis via sirtuin 1 regulation [124]. Consistent with these findings, fucoxanthin treatment significantly increased muscle mass in a dexamethasone-induced muscle atrophy mouse model [125]. Additionally, fucoxanthin treatment improves lipid peroxidation in the muscles and increases AMP-activated protein kinase (AMPK) phosphorylation. *Undaria pinnatifida* extract, which is rich in fucoxanthin, enhances mitochondrial biogenesis and increases the oxidative muscle fiber content in the skeletal muscle, leading to improved exercise capacity and muscle mass. Moreover, fucoxanthinol, a metabolite of fucoxanthin, possesses beneficial properties for age-associated sarcopenia [126]. It potentially combats sarcopenic obesity, as it inhibits H_2_O_2_-induced atrophy and loss in myotubes, activates lipolysis, and decreases the triglyceride content in mature adipocytes.

Astaxanthin, a xanthophyll carotenoid, possesses a notably high antioxidant capacity owing to its greater number of conjugated double bonds compared with numerous other carotenoids [127]. This pigment is found in various microorganisms and marine animals. Notably, *Haematococcus pluvialis* is a primary source for human consumption, and as a dietary supplement, astaxanthin is either extracted from *H. pluvialis* or obtained from seafood. Astaxanthin has been shown to improve mitochondrial function by activating AMPK in the skeletal muscle of high-fat-diet-fed mice [128]. In a tail-suspension-induced muscle atrophy mouse model, astaxanthin administration prevented mitochondrial dysfunction, alleviated mitochondrial oxidative stress and mitochondria-mediated apoptosis, and thus prevented muscle atrophy [129]. Furthermore, astaxanthin was revealed to improve cachexia, a muscle-wasting disease characterized by chronic inflammation, metabolic abnormalities, and hormonal changes, in mice with cancer cachexia [130]. The antioxidant effect of astaxanthin is more pronounced than that of other naturally occurring substances, which can prevent oxidative damage, a primary factor in the aging process. This substantial beneficial impact makes it a prominent subject of investigation in the field of aging research.

## 7. Considerations for Practical Applications

Marine organisms can be used in many applications due to the diversity of marine species. Marine organisms often possess weak physical defense mechanisms due to the unique ecosystems they inhabit. Consequently, their secondary metabolites tend to exhibit chemical properties distinct from those of terrestrial organisms [131]. Marine-derived secondary metabolites can be utilized not only as bioactive compounds, which are the focus of this study, but also as high-value compounds such as food, feed, and biofuels. Therefore, the study of biorefinery processes to obtain secondary metabolites from marine species should also be considered [132].

Microalgae are the representative example within biorefinery using marine sources. Microalgae can be developed into bioproducts through cultivation, harvesting, extraction, and application. Microalgae serve as valuable resources for bioactive compounds that could help prevent age-related diseases and support sustainable production [133]. For example, *Spirulina* and *Chlorella* spp. contain high levels of lipids and are used in biofuel research [134]. Alginate oligosaccharides derived from microalgae have the ability to be utilized as bioplastics or biopolymers, representing a sustainable alternative to address the issue of plastic pollution [135]. Furthermore, carbohydrates derived from algae can be fermented to produce bioethanol, which is emerging as a promising substitute for traditional energy sources [136,137].

However, there are some limitations to using marine sources as bioproducts, such as distinctive odors and off flavors [131], allergies [138], and hazardous contamination [139]. The distinctive odors and off flavors associated with marine organisms are perceived as limiting factors in their application as food additives or functional health products, primarily due to concerns regarding consumer acceptability [140]. To mitigate these issues, fish oils undergo hydrolysis and purification via enzymatic processes or microencapsulation [140,141]. In addition, allergies present significant challenges to the industrialization of marine resources, which are valuable in sectors like functional foods and health products [142]. At present, there is no known method to completely solve the problem of allergies. The development of non-allergenic alternatives that retain the bioactive properties of marine resources is a challenging and costly process [143]. Research related to marine allergies are focused on three main areas: the molecular identification of allergens, the improvement of diagnostic methods, and the development of immunotherapeutic agents. These approaches aim to alleviate allergies from marine sources [138].

Moreover, the use of marine products is associated with the presence of hazardous contaminants, particularly the accumulation of heavy metals. To achieve high-purity bioactive substances, it is essential to eliminate these contaminants [139]. Furthermore, the utilization of marine-derived bioactive substances is linked to the presence of toxic contaminants, such as heavy metal accumulation. The removal of these contaminants is essential for the extraction and utilization of bioactive substances derived from marine sources [144]. The use of bio-based methods employing specific lactic acid bacteria, including *Lacticaseibacillus rhamnosus*, can effectively mitigate the presence of marine toxins such as okadaic acid [145]. Furthermore, the incorporation of sorption premixes, such as vermiculite and perlite, has been demonstrated to significantly reduce lead toxicity in marine products by up to nine times [146]. The application of physical techniques, such as cold plasma and ultrasound, has also been shown to effectively control microbial contamination in fish products while simultaneously maintaining the quality and safety of the final products [144].

In most cases, marine species are composed of a multitude of compounds, and their purity is critical to harnessing the effects of a particular single compound. In order to extract and use specific compounds, research is required, such as innovative extraction methods in biorefinery technology. Furthermore, research is needed in the field of biorefinery processes in order to improve the above considerations for the utilization of marine sources.

## 8. Perspectives and Conclusions

As global demographics shift towards an increasingly older population, the demand for effective interventions to promote healthy aging and mitigate age-associated diseases has intensified. The aging process is inherently complex, influenced by genetic, environmental, and lifestyle factors, which collectively contribute to physiological, functional, and esthetic changes over time. Age-related diseases, such as CVD, metabolic disorders, neurodegenerative conditions, and musculoskeletal issues, pose significant challenges to healthcare systems worldwide. Bioactive compounds derived from marine sources offer innovative solutions to these challenges by targeting the underlying mechanisms of these diseases. Marine-derived bioactive compounds present a promising avenue for the development of functional foods designed to address a wide range of aging-related health concerns. This review highlights the nutritional and therapeutic potential of marine-derived compounds, examining their roles in supporting various physiological functions and preventing chronic disease.

Marine environments, which are abundant in unique bioactive compounds with potent biological activities, have historically been underutilized in the development of functional foods. These compounds, including antioxidants, omega-3 fatty acids, vitamins, minerals, and polysaccharides, have demonstrated significant potential in combating oxidative stress, inflammation, and metabolic disorders—key contributors to age-related health decline. For example, this review explores how marine-derived antioxidants, such as those found in seaweed and microalgae, protect cells from oxidative stress, a major factor in aging and various chronic diseases. Omega-3 fatty acids from marine sources, known for their cardiovascular benefits, are emphasized for their ability to reduce inflammation, stabilize atherosclerotic plaques, and improve endothelial function. Moreover, marine minerals and vitamins are examined for their contributions to bone health, immune function, and skin integrity. Incorporating these marine-derived compounds into functional foods offers a dual benefit: not only do they provide essential nutrients but they also deliver therapeutic effects that help safeguard the body’s natural defenses against aging. Given the rapid growth of the global anti-aging market, driven by technological advancements and increasing health awareness, particularly in North America, Europe, and Asia, the potential for marine-derived bioactive compounds in functional foods is substantial.

Bioactive compounds in functional foods can offer significant health benefits for older adults with aging-related diseases; however, careful consideration of their potential side effects is also necessary. Nutrient absorption issues may arise in older adults when consuming functional foods that are high in fiber. This is due to the fact that such foods can inhibit the absorption of essential minerals, including calcium and iron. Furthermore, certain polyphenolic compounds have been shown to reduce the bioavailability of iron and copper, potentially increasing the risk of anemia [147]. Additionally, the bioactive compounds present in functional foods may interact with prescription drugs, either enhancing or inhibiting drug metabolism, which could complicate the treatment of chronic diseases [148]. Overconsumption of bioactive compounds may lead to toxicity, and older adults may exhibit varying tolerances to these compounds, necessitating caution. Therefore, a balanced approach, guided by healthcare professionals, is essential for optimizing health outcomes in older adults.

The use of marine-derived bioactive compounds also has the potential to address a range of both biological and environmental issues. While this review primarily focuses on their role in combating age-related diseases, these compounds may also be applied to environmental challenges. For instance, fish skin and scales, often discarded as waste, can be repurposed as bioactive compounds to prevent skin aging, thereby addressing both waste management and enhancing material value. Additionally, marine pollution may be mitigated by replacing harmful chemicals used in industries such as agriculture and medicine with eco-friendly compounds derived from marine sources.

This review aims to identify the bioactive compounds derived from marine sources that can be safely ingested by humans and discuss their mechanisms of action and potential health benefits. Integrating these materials into functional foods has significant potential to improve the quality of life for aging populations, reduce the burden of age-associated diseases, and contribute to broader anti-aging research and health promotion. This perspective underscores the need for continued exploration and innovation in the use of marine-derived bioactive compounds, advocating for their extended application in functional foods as part of a holistic approach to promoting healthy aging.

## Figures and Tables

**Table 1:** Overview of marine sources, bioactive compounds, CVD-related diseases, and their biological effects.

Marine Source	Bioactive Compound	Target CVDs	Biological Effects	Ref.
Microalgae (*Dunaliella Salina)*	Zeaxanthin	Cardiac dysfunction	Elevates serum levels of homocysteine, creatinine kinase isoenzymes, and lactate dehydrogenase	[30]
Seaweed	Fucoxanthin	Heart valve disease	Decreases oxidative-stress-induced apoptosis and modulates Akt/ERK-related protein expression	[31]
Algae (*Sargassum fusiforme*)	Saringosterol	Atherosclerosis	Activates liver X receptors α and β to regulate cholesterol levels	[32]
Fungi (*Aspergillus* sp.)	Asperlin	Atherosclerosis	Reduces pro-inflammatory factors and decreases levels of iNOS, IL-1β, and TNFα expression	[33]
Marine sponge (*Acanthostrongylophora ingens)*	Manzamine A	Atherosclerosis	Decreases the total levels of free and LDL cholesterol and triglycerides	[34]
Algae (*Haematococcus pluvialis*)	Astaxanthin	Atherosclerosis	Decreases the total levels of triglyceride and cholesterol	[35]
Fungi (*Amphichorda feline*)	Isaridin E	Atherosclerosis	Downregulates the PI3K/Akt signaling pathway and has anti-inflammatory and anti-thrombotic effects	[36]
Brown algae	Alginate oligosaccharides	Hypertension	Decrease the expression of P-selectin and inhibit the p38MAPK/NF-κB pathway	[37]
Mangrove fungi	Xyloketal B	Atherosclerosis, hypertension, cardiac stroke	Promotes endothelial NO release, regulation of the Akt/eNOS pathway, and reductions in oxidative stress and has an antihypertensive effect	[38,39]
Fish oil	Omega-3 fatty acids (EPA and DHA)	Atherosclerosis, myocardial infarction, cardiac arrhythmia	Reduce inflammation, lower blood pressure, and improve lipid profiles	[40]

## Data Availability

The datasets used are available on request from the authors.
